# Deep learning-based detection of indicator species for monitoring biodiversity in semi-natural grasslands

**DOI:** 10.1016/j.ese.2024.100419

**Published:** 2024-04-09

**Authors:** Deepak H. Basavegowda, Inga Schleip, Paul Mosebach, Cornelia Weltzien

**Affiliations:** a Technische Universität Berlin, Straße des 17. Juni 135, 10623, Berlin, Germany; b Leibniz-Institut für Agrartechnik und Bioökonomie e.V. (ATB), Max-Eyth-Allee 100, 14469, Potsdam, Germany; c Hochschule für nachhaltige Entwicklung Eberswalde, Schicklerstraße 5, 16225, Eberswalde, Germany

**Keywords:** Species-rich grasslands, Biodiversity monitoring, Object detection, Cross-domain knowledge transfer

## Abstract

Deep learning (DL) has huge potential to provide valuable insights into biodiversity changes in species-rich agricultural ecosystems such as semi-natural grasslands, helping to prioritize and plan conservation efforts. However, DL has been underexplored in grassland conservation efforts, hindered by data scarcity, intricate ecosystem interactions, and limited economic incentives. Here, we developed a DL-based object-detection model to identify indicator species, a group of vascular plant species that serve as surrogates for biodiversity assessment in high nature value (HNV) grasslands. We selected indicator species *Armeria maritima, Campanula patula, Cirsium oleraceum,* and *Daucus carota*. To overcome the hurdle of limited data, we grew indicator plants under controlled greenhouse conditions, generating a sufficient dataset for DL model training. The model was initially trained on this greenhouse dataset. Then, smaller datasets derived from an experimental grassland plot and natural grasslands were added to the training to facilitate the transition from greenhouse to field conditions. Our optimized model achieved remarkable average precision (AP) on test datasets, with 98.6 AP_50_ on greenhouse data, 98.2 AP_50_ on experimental grassland data, and 96.5 AP_50_ on semi-natural grassland data. Our findings highlight the innovative application of greenhouse-grown specimens for the in-situ identification of plants, bolstering biodiversity monitoring in grassland ecosystems. Furthermore, the study illuminates the promising role of DL techniques in conservation programs, particularly as a monitoring tool to support result-based agri-environment schemes.

## Highlights

• Lack of data impedes deep-learning applications for grassland monitoring.
• We solve the data scarcity problem using a greenhouse-based data pipeline.
• We train an object detector specifically for *in situ* plant identification.
• We align data distribution between greenhouse and grassland domains via bridge data.
• Different morphological features are essential to distinguish similar plants.

## Graphical abstract

**Figure:**
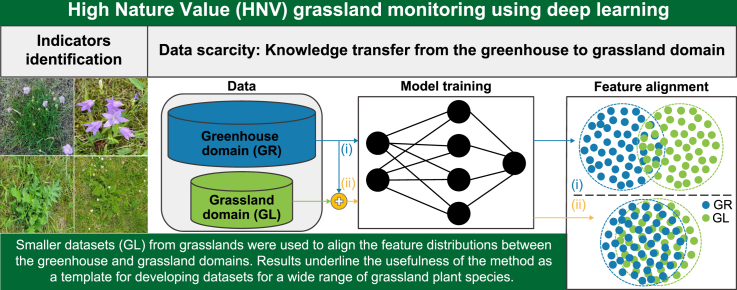

## 1 Introduction

Semi-natural grasslands are among the most species-rich ecosystems in the world [1] and accommodate a wide range of flora and fauna, including numerous endangered species [[2], [3], [4], [5]]. These species-rich grasslands provide various ecosystem services, such as carbon sequestration, climate mitigation, pest control, pollination, soil fertility, and water regulation [4,6,7]. Therefore, they play an essential role in human well-being. In Europe, these species-rich ecosystems cover 34% of the European agricultural landscape [8] and have a more significant role in preserving biodiversity and delivering ecosystem services. However, since the 1950s, these species-rich habitats have steadily declined, quantitatively and qualitatively, primarily due to current agricultural practices and land use changes such as intensification, urbanization, abandonment, afforestation, and conversion to cropland [9].

The widespread decline of semi-natural habitats results in a greater biodiversity loss and emphasizes the importance of extensive, low-input, and often traditional farming practices that promote a more diverse and resilient ecosystem through sustainable and environmentally friendly land use [[10], [11], [12]]. The importance of this extensive and low-input farming was acknowledged in Europe in the early 1990s [13]. These practices create habitats for wildlife and support a wide range of species, such as pollinators, birds, and soil organisms, leading to more sustainable agriculture [10]. Simultaneously, conservation actions that halt, slow, or reverse biodiversity loss can significantly slow anthropogenic-mediated climate change [14]. In this context, the high nature value (HNV) farmland concept was developed to recognize agricultural landscapes with high levels of biodiversity and habitats of conservation concern [11,13].

Species-rich grasslands are an important component of HNV farmland and are among the habitats most in need of conservation in Europe [4]. Therefore, several conservation programs have been established to conserve these species-rich habitats [15]. To evaluate the output of these conservation programs, some indicators, such as the HNV farmland indicator, have been proposed and put forward by the European Union's rural development policy framework [16]. These indicators serve as metrics for assessing quantitative and qualitative changes in HNV farmlands. There are no universally applicable rules to derive HNV farmland indicators, as each European country decides how to derive them.

***HNV grassland monitoring***. In Germany and other European countries, a list of vascular plants called HNV indicator or character species is used to identify and monitor HNV grasslands [17]. In general, this group of organisms does not represent all grassland biodiversity, but correlations between plant richness and the richness of other organisms, such as wild pollinators, grasshoppers, and gastropods, could be evidenced [18,19]. According to this approach, the number of indicator species identified on transect segments must meet or exceed a threshold value for grassland to be characterized as HNV grassland. Based on the number of occurring indicator species, the semi-natural grassland is then assigned to one of three quality levels: extremely high nature value (HNV I), very high nature value (HNV II), and moderately high nature value (HNV III) [20]. Grasslands not meeting these minimum criteria are classified as grasslands with no HNV.

The standard method to monitor HNV grasslands involves periodically conducting field surveys on a selected set of grassland sites, where plant experts visually identify indicator plants on transects and consequently update the HNV status [20]. However, these field-based surveys are typically tedious, time-consuming, cost-intensive, and suffer from poor spatial and temporal sampling. They may also contain a notable degree of uncertainty due to potentially missed species [[21], [22], [23]]. The quality of these surveys depends mainly on the taxonomic expertise and performance of the surveyors. In addition to these drawbacks, the traditional approach to grassland monitoring is further complicated by the sheer size of the monitoring sites and the fact that measurements must be taken periodically. Therefore, developing an autonomous system for identifying indicator species can contribute significantly to a detailed understanding of grassland ecosystem structure and function. Furthermore, an autonomous tool to identify indicator species can be handy in assisting result-based agri-environment schemes (AES), as monitoring indicator species is one of the significant barriers to the widespread implementation of result-based AES [24,25].

***Deep learning (DL) applications in* grasslands**. Unlike traditional machine learning, DL methods can recognize complicated structures in high-dimensional data with minimal human intervention [26,27]. For this reason, DL models have become the first choice for many machine vision tasks, where they rely on trainable computational modules consisting of a potentially large stack of processing layers to extract information patterns from input data. During training, each layer learns a more abstract representation of the data based on the more elementary representation in the previous layer. This type of learning allows the model to recognize and extract features from the input data automatically. Object detection is a fundamental machine vision task for locating instances of interested objects in images and videos. Object detection based on DL can be split into two tasks: object classification to predict and assign interested objects to targeted classes and object localization to estimate the spatial location and the extent of each detected object.

DL methods are widely used in agriculture to identify and characterize plant species [[28], [29], [30], [31], [32]], but most of these studies focus on arable and orchard crops, while few efforts for grassland species can be found in the literature [33,34]. Several studies have used spectral information and other spatial data to distinguish HNV areas from non-HNV areas by identifying species communities [[35], [36], [37]]. However, the studies also conclude that identifying indicator species at the plant level, and thus of HNV classes, is hardly possible based on spectral information alone [36]. Our study addresses this research gap by developing an object detection model based on DL to monitor HNV grasslands and assess their quality levels by detecting indicator species.

In this context, the main objectives of the present study are to (1) explore the potential of DL to monitor semi-natural grasslands at the species level by reviewing the current status and outlining associated challenges, (2) develop a cost-effective data pipeline to generate training data for indicator species to address the data scarcity problem, and (3) develop an object detection model to address objective (1) and to validate objective (2).

## 2 Materials and methods

### 2.1 Selection of indicator plants

**Fig. 1:**
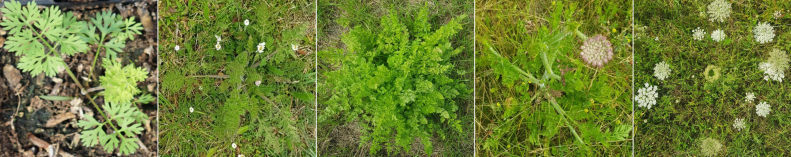
Example images of *Daucus carota* showing different growth stages from the early vegetative stage to the late flowering stage.

For this study, we selected four HNV indicator species: *Armeria maritima*, *Campanula patula*, *Cirsium oleraceum*, and *Daucus carota*. The species are from the HNV indicator list of the Northeast region of Germany, which includes the German states of Mecklenburg-Vorpommern and Brandenburg. The species were selected based on the following criteria: they are relatively common in semi-natural grasslands in the Northern Brandenburg region, ensuring feasible access to naturally grown plants for validation purposes; and they exhibit distinct and diverse morphological features, which introduce varying degrees of detection difficulty during the vegetation period. In addition, individuals of the same species can exhibit significant variation in appearance depending on their age, growth, and environmental factors (Fig. 1).

*Armeria maritima* is one of those species in grasslands that is potentially very difficult to identify because of its narrow, grass-like leaves. *Campanula patula* is a small-sized species with undivided and narrow leaves. *Cirsium oleraceum* is a broad-leaved species with a very characteristic leaf shape. It is distinct from grasses and other small-leaved herbs, making it easier to spot in the sward. *Daucus carota* has multi-pinnate leaves, a typical feature of Apiaceae*. Campanula patula* and *Daucus carota* represent a category of supposed medium difficulty. The four species have different ecological demands: *Armeria maritima* is a species of dry grasslands, *Campanula patula* and *Daucus carota* are typical species of semi-dry grasslands, and *Cirsium oleraceum* is a typical species of moist grasslands.

### 2.2 Data

**Fig. 2:**
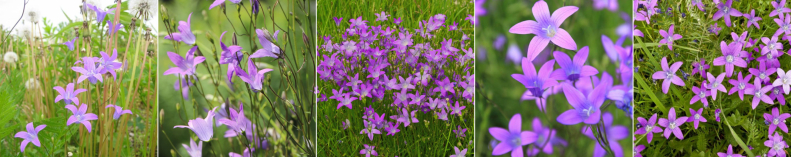
Example images of *Campanula patula* collected from the Global Biodiversity Information Facility (GBIF) database showing a strong bias towards the flowering period with skewed view characteristics.

Developing an autonomous plant identification system for grassland species faces numerous challenges, mainly due to the scarcity of large, annotated image datasets and the suitability of the existing data. For example, the Global Biodiversity Information Facility (GBIF) provides free and open-access biodiversity data, including many grassland plant species [38]. The data, contributed by various institutions and individuals globally, is made readily available and searchable via the GBIF portal through its extensive infrastructure. However, images collected from the GBIF portal have limitations, such as a strong bias for a particular growth phase (flowering stage) and skewed front-view characteristics (Fig. 2). The study focused on identifying indicator species during both the vegetation and flowering periods and aimed to collect data from the nadir perspective so that the data could be used in future work to detect indicator species using unmanned aerial vehicles (UAVs).

To address the data scarcity problem, we propose a method based on training an object detection model principally on greenhouse data, i.e., growing indicator species in a greenhouse to prepare an adequate amount of image data and adapting the model trained on this greenhouse data to detect naturally grown plants using a relatively small amount of data from grasslands. First, we considered the greenhouse approach to develop image datasets for the indicator species due to the difficulty in finding and collecting these species in adequate quantities from grasslands. Indicator species serve as proxies of high biodiversity in grasslands, which is declining dramatically. Furthermore, the structural complexity of grasslands and the canopy architecture of grassland plant communities make it difficult to generate good training data, especially for lower canopy species. Second, the indicator species were planted on an experimental grassland plot to bridge the gap between the model trained on greenhouse plants and plants grown under natural conditions. We also collected images of naturally grown indicator plants from various semi-natural grasslands to evaluate the method against real data.

#### 2.2.1 Greenhouse data

**Fig. 3:**
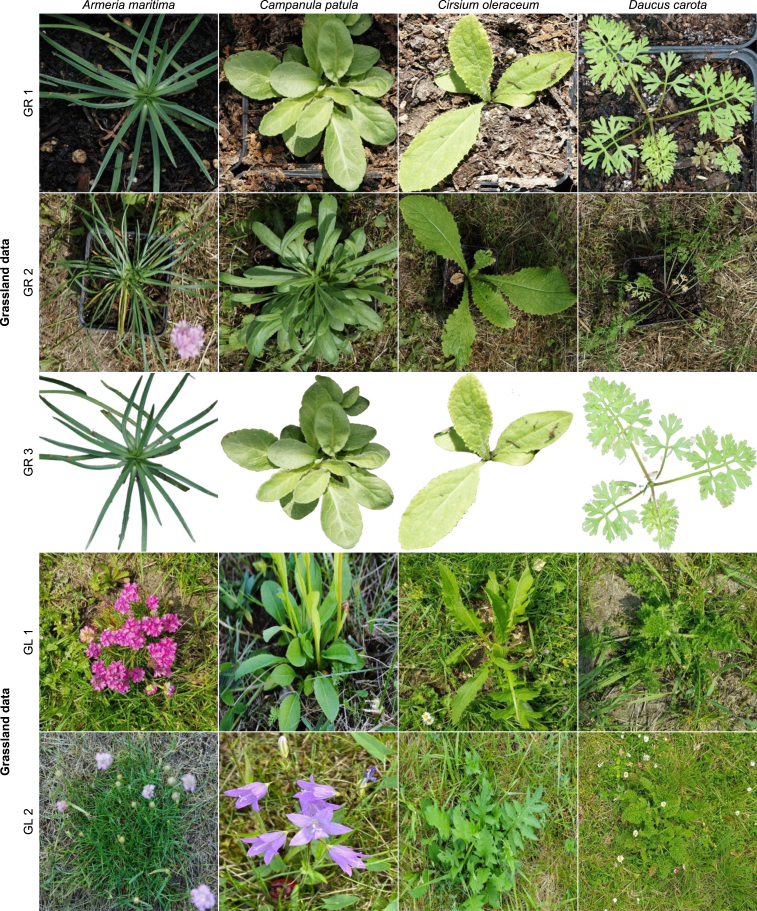
Greenhouse data: Images collected in the greenhouse (GR 1) and outside on a meadow (GR 2) and background-removed images (GR 3). Greenhouse data accounted for about 70% of the total images collected. Grassland data: Images collected from the experimental grassland plot (GL 1) and semi-natural grasslands (GL 2).

The greenhouse facility at the Leibniz Centre for Agricultural Landscape Research (ZALF), Müncheberg, Germany, was used for the greenhouse data collection approach. Seeds of the selected indicator species were sown in plant pots during the second week of April 2020. Two hundred plant pots were used for each indicator species. Twelve data collection campaigns were conducted from May 2020 to September 2020 with two experimental setups. In the first experimental setup, images of indicator plants were acquired in the greenhouse. In the second experimental setup, images were acquired outside the greenhouse by randomly spreading plants on a meadow (Fig. 3).

The images were acquired using a terrestrial system (manually driven) constructed using aluminum frames mounted on four bike wheels. It was designed to adjust the mounting point of an image sensor from 1 to 2 m above the ground. Images were acquired using a 24-megapixel consumer-grade camera (α-6000, Sony, Tokyo, Japan) with an APS-C type sensor chip (23.5 × 15.6 mm). The sensor was mounted in the nadir position to capture top-down view images. The sensor had a resolution of 6000 × 4000 pixels. Images were captured autonomously with one image per second frequency as the system moved over greenhouse plants.

#### 2.2.2 Background subtraction

Images acquired inside the greenhouse contained unrealistic background data, i.e., plant growing substrate and the greenhouse floor tiles. Naturally grown plants usually have grass and other vegetation as background. Therefore, background subtraction techniques were applied to remove the background on images collected in the greenhouse. The background-removed images were included in the model training to investigate the impact of unrealistic background data on the model's performance. In order to remove background data, images were first converted from the RGB (red, green, and blue) colorspace into the HSV colorspace (hue, saturation, and value). The HSV colorspace separates the intensity (brightness) from the chromaticity (color information) and represents them individually. The separation results in better foreground object segmentation than other color models [39].

After the HSV color filtering, the Canny edge detector with morphological transformation was applied to remove the remaining image noise. Edge detection is an image processing technique used to distinguish the boundaries of objects within images by finding the points where the brightness of pixels changes significantly. In a given image, the Canny edge detector [40] applies a Gaussian kernel to smooth noise and then computes the edge gradient and direction for each pixel in the smoothed image. After finding the intensity gradient, a full image scan is performed to identify the candidate edge pixels and remove any unwanted pixels that may not constitute the edge through an edge-thinning process called non-maximal suppression.

#### 2.2.3 Grassland data

For the experimental grassland approach, indicator plants were grown on a grassland plot at the research station of the Leibniz Institute for Agricultural Engineering and Bioeconomy (ATB, Potsdam) in Marquardt, Potsdam, Germany. The indicator species *Armeria maritima, Cirsium oleraceum,* and *Daucus carota* were planted between the third week of April 2022 and the second week of May 2022 in a grassland plot of size 20 × 30 m, with a count of fifty plants per species. Images were captured manually between April 2022 and August 2022 in ten different data collection campaigns using a cell phone camera with an image resolution of 2160 × 2160 pixels. The images were taken from the nadir view. Unfortunately, we only had limited data available for *Campanula patula* from the experimental plot and the semi-natural grasslands to include them in the model training. Therefore, *Campanula patula* was omitted from the grassland training data.

Images of naturally grown indicator plants (Fig. 3) were collected from different grassland sites around Eberswalde and Potsdam, Germany, to evaluate the developed approach against real data. The data contained *Armeria maritima*, *Cirsium oleraceum*, and *Daucus carota* images. Images were taken manually using a Sony α-6000 camera with an image resolution of 6000 × 4000 pixels and a cell phone camera with an image resolution of 2160 × 2160 pixels.

In summary, the datasets contain (i) Greenhouse data (GR 1, GR 2, and GR 3): time-series images of the indicator plants collected from the greenhouse, and (ii) Grassland data (GL 1 and GL 2): time-series images of the indicator plants collected from the experimental grassland plot and images of naturally grown indicator plants collected from various grassland sites (Table 1). Images collected using greenhouse-grown plants accounted for a large proportion of the data collected, representing more than 70% of the total data collected.

**Table 1:** Overview of indicator species data used in the study. The numbers represent the image instances of each indicator species under different datasets.

Dataset name	*Armeria maritima*	*Campanula patula*	*Cirsium oleraceum*	*Daucus carota*
GR 1: Greenhouse plants collected in the greenhouse	825	984	632	696
GR 2: Greenhouse plants collected outside on a meadow	480	670	370	545
GR 3: Background subtraction applied on GR 1	810	890	625	580
GL 1: Plants collected from the experimental grassland	462	-	332	562
GL 2: Naturally grown plants from grasslands	478	-	568	525

### 2.3 Object detection

#### 2.3.1 EfficientDet

For this work, a single-stage object detection model based on the EfficientDet architecture was selected to develop a real-time detection model with higher accuracy [41]. EfficientDet models are a family of scalable and efficient object detectors. These models achieved state-of-the-art accuracy while being smaller and using significantly less computational power than other prior state-of-the-art detectors. The compound scaling method used in their architecture enables different model scaling and selection to build an adequate object detector for a wide range of resource constraints from mobile devices to data centers, with a uniform scaling on the resolution, depth, and width of the neural network components. EfficientDet architecture has three main components: EfficientNet, BiFPN or Weighted Bi-directional Feature Pyramid Network, and class/box network. In summary, EfficientNet [42] extracts features from an input image. BiFPN outputs a list of fused features representing salient features of the input image by taking multiple levels of features as input. Finally, a class/box network is used to predict the class and location of each object from the fused features.

#### 2.3.2 Loss functions

Loss functions are quantitative measures to assess the accuracy of model predictions against the ground-truth annotations. In object detection, losses are mainly categorized into classification loss and regression or localization loss. Classification loss is used to measure the model's performance in predicting the true class of the detected objects, and regression loss is used to measure the model's performance in predicting the spatial location of the detected objects in a given image. It is measured by applying intersection over union (IoU) between the predicted and ground-truth bounding boxes. Therefore, the total loss is a sum of classification and localization loss for object detection tasks.

Focal loss [43] is commonly used to measure classification loss. It is designed to address the extreme imbalance problem between foreground and background classes, which generally affects the performance of single-stage object detectors, during training by focusing on hard examples that the model incorrectly predicts rather than on those it can confidently predict. Focal loss handles this problem by modifying the standard cross-entropy (CE) loss to focus training on a sparse set of hard examples. The modified CE loss is given by:

(1) $$\text{CE}\left(p,y\right)=\left\{ \begin{array}{l} -\;\alpha \;\log\left(p\right)\;\text{if}\;y=1\\ -\;\left(1-\;\alpha \right)\log\left(1-p\right)\;\text{otherwise} \end{array}\right.$$

where y∈{±1} specifies the ground-truth class, p∈[0,1] is the model's estimated probability, and α∈[0,1] is a weighting factor. In Equation (1), α balances the importance of positive and negative examples, but it does not differentiate between easy/hard examples. Therefore, authors [43] proposed to add a modulating factor ${\left(1-p\right)}^{\gamma }$ to the modified CE loss in order to downweight easy examples and focus training on hard examples, with a tunable focusing parameter γ≥0. The modulating factor reduces the loss contribution from easy examples and increases the importance of correcting misclassified examples [43]. Focal loss (FL) is given by:

(2) $$\text{FL}\left(p\right)=\left\{ \begin{array}{l} -\;\alpha \;{\left(1-p\right)}^{\gamma }\;\log\left(p\right)\;\text{if}\;y=1\\ -\;\left(1-\;\alpha \right)p^{\gamma }\;\log\left(1-p\right)\;\text{otherwise} \end{array}\right.$$

Focal loss (FL) was used to calculate the classification loss, and smooth $L_{1}$ or Huber loss was used to calculate the regression loss [44].

#### 2.3.3 Evaluation metrics

The standard outputs of the object detection models are the predicted classes and the spatial locations of the detected objects with bounding box coordinates. Average precision (AP) is a widely used metric for evaluating the performance of object detection models. For calculating AP, a threshold value for IoU is first set to distinguish between true positive (TP), false positive (FP), and false negative (FN) detections. IoU is the ratio of the area of overlap to the area of union*,* $\frac{b\cap b_{g}}{b\cup b_{g}}$, where *b* is the area of the predicted bounding box, and *b*_*g*_ is the area of the ground-truth bounding box. For detection to be TP, the predicted class *c* must match the ground-truth class *c*_*g,*_ and the IoU value must be greater than the threshold value. Otherwise, the prediction is considered FP. FN is counted for the missing detection of any ground-truth objects. Precision (P) indicates what proportion of the positive identification was actually correct, $\frac{TP}{TP+FP}$. Recall (R) indicates what proportion of actual positives correctly identified, $\frac{TP}{TP+FN}$. Then, average precision (AP) is calculated by averaging the precision values over recall values ranging from 0 to 1.

(3) $$\text{AP}=\int_{R=0}^{1}P\left(R\right)dR$$

AP was calculated according to the COCO (common objects in context) object-detection evaluation standards [45], where precision is averaged over recall values uniformly distributed from 0 to 1 with a step size of 0.01. AP at an IoU of 0.50, 0.05, and 0.95*,* the standard metric, corresponds to averaging AP over ten different IoU thresholds between 0.5 and 0.95 with a step size of 0.05 across all classes. Traditionally, this is called mean average precision (mAP). For calculating AP at a specific IoU value, the area under the precision-recall curve for that specific IoU value is considered. By setting the IoU threshold, a metric can be more or less restrictive in considering detections as correct or incorrect. For example, AP^IoU = 0.50^ (AP_50_) counts detections at IoU values of 0.5 and above as correct, and AP^IoU = 0.75^ counts detections at IoU values of 0.75 and above as correct, with a perfect match occurring at IoU = 1.

### 2.4 Implementation details

Images were annotated using LabelImg, an open-source image annotation tool. The object detection model was optimized using a stochastic gradient descent (SGD) optimizer with a momentum of 0.9 and weight decay of 4 × 10^−5^. The learning rate was linearly increased from 0 to 0.05 in the first epoch and then annealed down using the cosine decay rule. The focal loss function is employed with a focusing parameter (γ=1.5) and weighting factor (α=0.25). The model was trained for 300 epochs with a total batch size of 12 on four NVIDIA GeForce RTX 2080Ti GPUs, each with 11 GB of memory. TensorFlow 2 and Python 3 were used for the implementation.

## 3 Results

### 3.1 Greenhouse and grassland domain

In training, datasets were considered under two domains: the greenhouse domain with a sufficiently large number of data samples and the grassland domain with relatively less data. Data from the greenhouse domain represented a major portion of the training data, and the model was principally trained on this data and applied to detect naturally grown indicator plants with the knowledge learned from the greenhouse-grown plants. We used experimental grassland data as an intermediate (bridge) domain to align the data distribution between the greenhouse and grassland domains.

**Fig. 4:**
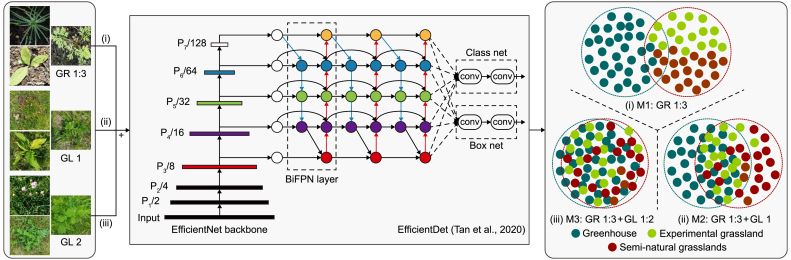
Overview of the knowledge transfer between the greenhouse and grassland domains. The model was principally trained on greenhouse data and applied to detect naturally grown indicator plants in grasslands. Images from the experimental grassland plot were used as an intermediate domain to align the data distribution between the greenhouse and grassland domains in training.

The use of data samples from the greenhouse domain to detect plants in the grassland domain through knowledge transfer is shown in Fig. 4. We conducted three experiments to demonstrate the knowledge transfer between the domains. The model was trained with a combination of different datasets for each experiment: (i) exclusively using greenhouse data, (ii) a combination of greenhouse and experimental grassland data, and (iii) a combination of greenhouse, experimental grassland, and semi-natural grassland data. We reserved 20% of data from each source as test data to validate the knowledge transfer between the domains. *Campanula patula* was excluded from experiments 2 and 3 due to insufficient data samples from the experimental grassland and semi-natural grasslands. Table 2 summarizes the performance of the models evaluated on test datasets under three different experimental setups.

**Table 2:** Evaluation results of the experiments. The models were evaluated on the test datasets from the greenhouse, the experimental grassland, and semi-natural grasslands. The significant increase in model performance after adding experimental and grassland data demonstrates the knowledge transfer between the greenhouse and grassland domains.

Experiments		Greenhouse data (GR 1:3)		Experimental grassland data (GL 1)		Semi-natural grasslands data (GL 2)		Overall test data	
No.	Trained on	AP	AP_50_	AP	AP_50_	AP	AP_50_	AP	AP_50_
Model 1 (M1)	Greenhouse data	**79.7**	**97.1**	8.7	21.6	27.1	44.1	79.7	97.1
Model 2 (M2)	Greenhouse and experimental grassland data	79.7	97.8	**62.6**	**96.3**	45.3	71.9	77.6	97.6
Model 3 (M3)	Greenhouse, experimental grassland, and natural grasslands data	82.0	98.6	66.0	98.2	**65.4**	**96.5**	79.4	98.5

Experiment 1 was conducted to compare the performance of the model (M1) against naturally grown indicator plants, where grassland data was not included in the training. The model evaluated on test datasets achieved 79.7 AP and 97.1 AP_50_ on greenhouse data, 8.7 AP and 21.6 AP_50_ on experimental grassland data, and 27.1 AP and 44.1 AP_50_ on natural grassland datasets. Compared to greenhouse data, the model's poor performance against grassland data indicates a distribution change or domain shift between greenhouse and grassland datasets. The difference in the model's performance between experimental and semi-natural grassland datasets shows that experimental grassland data was a less general subset of the latter.

Experiment 2 was conducted to observe the change in performance of the model (M2) when experimental grassland data, which accounted for 15% of the training data, was added to the model training. We did not observe any significant performance change against the greenhouse data. The model's performance on the experimental grassland data improved significantly, with the detection performance improving from 8.7 to 62.6 AP and from 21.6 to 96.3 AP_50_, indicating that the addition of experimental grassland data to the model training aligned the data distribution between the domains. Although the AP values differed noticeably between greenhouse and experimental grassland data, the AP_50_ values were nearly identical. This difference highlights the complexity of the grassland domain compared to the greenhouse domain for the localization task.

The improved performance on semi-natural grassland data from 27.1 to 45.3 AP and 44.1 to 71.9 AP_50_ highlights the potential of using greenhouse plants to detect grassland plants and the usefulness of bridge data in aligning the data distribution between greenhouse and grassland domains. This change in performance is particularly noteworthy, considering semi-natural grassland data was not included in the model (M2) training. Although this experiment indicates the knowledge transfer from the greenhouse to the grassland domain, the model's performance against the natural grassland data was not on par with the experimental grassland data. The third experiment was conducted to observe the further alignment between the greenhouse and grassland domains.

For experiment 3, the model (M3) was trained by combining the greenhouse, experimental grassland, and semi-natural grassland datasets, with the latter two contributing about 30% of the training data. The model's performance on greenhouse and experimental grassland data improved slightly. The model's performance on natural grassland data improved significantly from 45.3 to 65.4 AP and 71.9 to 96.5 AP_50_. The increase in the model's performance on all datasets indicates further alignment between the domains.

### 3.2 Impact of background subtraction

**Fig. 5:**
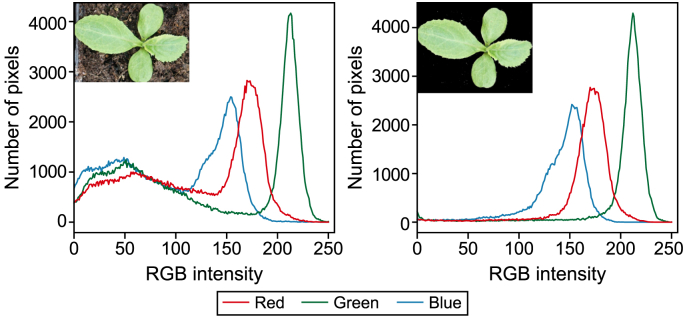
Histogram of an image with (left) and without (right) background data. Adding background-subtracted images to the training improved the overall model performance.

To investigate the impact of unrealistic background data, i.e., plant growing substrate, on the model training, we evaluated the model [M1] before adding background-subtracted images. Fig. 5 shows the histogram comparison for a greenhouse plant with and without the background data. The model achieved 73.3 AP and 96.9 AP_50_ on greenhouse test data before adding background-subtracted images. By adding background-subtracted images, we observed an improvement in performance from 73.3 to 79.7 AP and 96.9 to 97.1 AP_50_ on greenhouse data and from 3.8 to 8.7 AP and 11.5 to 21.6 AP_50_ on experimental grassland data. Similarly, when evaluated on semi-natural grassland data, the performance improved from 21.5 to 27.1 AP and 33.0 to 44.1 AP_50_. The background-subtracted images were not used in the evaluation of the model. This comparison shows that the unrealistic background information in the source domain (greenhouse) negatively affected the detection in the target domain (grassland).

### 3.3 Indicator species

As mentioned in section 2.1, the indicator species were selected based on their distinct characteristics and diverse morphological features. Therefore, the comparison of the model's performance on each species reflects the difficulty of their detection. We used the optimized model (M3) for the comparison and excluded greenhouse data because the model performed equally well on greenhouse data for all the indicator species. The model was evaluated on each species using the experimental grassland and semi-natural grassland data.

**Fig. 6:**
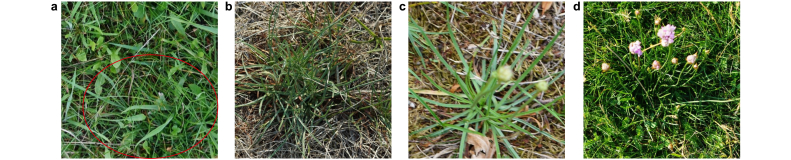
**a**, *Armeria maritima* is very hard to detect when it occurs in a homogeneous blend with grass. The red circle indicates the presence of *Armeria maritima*. **b**–**d**, Additional features such as the background distinction between a plant and the grass, as shown in panels **b** and **c**, and the presence of flowers panel **d** are instrumental in the identification of such species.

*Armeria maritima* presents the greatest challenge for detection among the four species studied, primarily due to its grass-like leaves. Adding images of *Armeria maritima* from the vegetation period to the model training increased the false positive detections, i.e., the recognition of grass as *Armeria maritima*. Therefore, we only trained the model with images of *Armeria maritima* that have additional features such as flowers and background distinction (Fig. 6). The optimized model (M3) achieved 61.3 AP on *Armeria maritima*, 72.1 AP on *Cirsium oleraceum*, and 63.5 AP on *Daucus carota*.

## 4 Discussion

The performance of the optimized model (M3) evaluated on grassland data, particularly on naturally grown indicator plants, demonstrates the merit of our approach in addressing data scarcity for grassland plant species. The results in Table 2 show that the model's performance is comparable between greenhouse and grassland data when the AP_50_ metric is used for the evaluation. However, the grassland data contained certain reservations, such as excluding *Armeria maritima* during its vegetative period. The AP_50_ evaluation results indicated the ability of the model to do the classification task, i.e., to identify the true class of the detected indicator species without determining the exact spatial position. On the other hand, the standard AP, which is more indicative of the ability of an object detection model to determine the exact spatial position, showed the complexity of grassland data for the localization task compared to greenhouse data. The difference in results between the grassland and greenhouse domains is expected for two reasons: grassland data is highly complex compared to greenhouse data and represents only 30% of training data. Incorporating additional grassland images could improve the standard AP on grassland data; however, it is unlikely to match the AP levels achieved with greenhouse data.

The model's performance against individual species is consistent with the species description in section 2.1. The results show that *Armeria maritima* is more difficult to identify during the vegetative period than other species. This is analogous to manual identification, where *Armeria maritima* plants are more difficult to identify in swards during vegetative periods unless examined very closely. Adding such images of *Armeria maritima* to the training data will have a negative impact on performance, as a high false positive rate will result from the misidentification of grass as *Armeria maritima*. Even annotating such images is arduous when no ground information from the field exists. Therefore, additional morphological characteristics of the plants are essential features for deep learning models to distinguish this kind of species from the background. For example, we considered images of *Armeria maritima* with flowers and a distinct background to train the model (Fig. 6).

Adding such additional features indicates the importance of measuring some species at certain times of the year, such as during the flowering period. With this constraint, the model's performance on *Armeria maritima* is on par with *Daucus carota*. The crowd-counting problem [46] affected the model's performance on *Daucus carota*, i.e., the high density of overlapping plants, which is also true to some extent for other species. The performance of the object detector is higher for *Cirsium oleraceum*, proving that this species is easier to identify than other species due to its broad leaf characteristics. The standard AP is also higher for *Cirsium oleraceum*, suggesting that localization is specific to species along with classification. Since distinctive species were selected, we did not observe any major interspecific misclassification. For example, interspecific misclassification between *Daucus carota* and *Conium maculatum* could be higher.

In this study, we grew indicator plants in the greenhouse to acquire image data for training DL models, given that growing plants and collecting images in a controlled environment is easier and less expensive than in grasslands. Our approach is based on the hypothesis that greenhouse plants could facilitate the detection of naturally occurring plants in grasslands, as the greenhouse and grassland domains share plant-specific features. Although they share a common feature space [47], there is a domain shift to directly apply the knowledge learned from the greenhouse domain to detect plants in the grassland domain. We used an intermediate domain in which experimental grassland images were used to align features between the greenhouse and grassland domains, thus transferring knowledge from the data-rich greenhouse domain to the data-poor grassland domain. The performance gain of the model (M2) validated our hypothesis, with the average precision rising from 44.1 to 71.9 AP_50_ on semi-natural grassland data after the addition of experimental grassland data to the model training.

The greenhouse and experimental grassland data represented time-series images of the indicator species from the early vegetative stage to the late flowering stage. However, greenhouse data did not contain adequate images of plants with flowers and flower buds. Data from semi-natural grasslands contained images from the vegetative and flowering periods but did not contain time-series images of indicator species. *Campanula patula* was included only in experiment 1, as there were insufficient data samples from the experimental grassland and semi-natural grasslands. In addition, we did not comprehensively apply deep domain adaption methods to solve the domain shift problem between the greenhouse and grassland domains. Applying such methods may improve the model's performance while reducing the data required from the grassland domain [39,47].

## 5 Conclusion and outlook

Understanding the drivers of biodiversity loss is critical to the success of conservation efforts, which requires continuous monitoring of targeted areas. Technological innovations in sensors and sensing platforms offer cost-effective ways to collect near-continuous data over a large number of sites. While these tools are helping to develop more accessible and affordable biodiversity data, they also result in an ever-increasing data glut. For these tools to be more effective, automated approaches must be coupled to process and analyze collected data to derive meaningful information.

In this work, an object detection model was developed to detect HNV indicator species to demonstrate the potential of DL for monitoring biodiversity in high-ecological value grasslands, such as HNV grasslands. At the beginning of the study, we noted that one of the biggest obstacles to the limited research on developing DL applications for grassland is the lack of extensive annotated datasets. Given the cost and challenges associated with developing high-quality datasets for grassland species, we developed a data collection method based on greenhouse-grown plants. The change in the data distribution, called domain shift, between greenhouse- and naturally-grown plants was reduced using bridge data, i.e., datasets containing images of indicator plants grown on the grassland plot with a similar feature space as naturally-grown plants. Adding bridge data to the model training aligned the data distribution between the greenhouse and grassland domains. The results of the models (M2 and M3) on naturally grown plants verify the knowledge transfer from the greenhouse domain to the grassland domain. The results also underline the usefulness of our approach as a template for developing datasets for various grassland plant species.

In summary, the study has demonstrated the potential of DL to assist in monitoring semi-natural grasslands by identifying HNV indicator species. However, more indicator species need to be added to the training database to make it operational. We selected a few indicator species to show how the data scarcity problem for grassland plant species can be solved. At the landscape level, identifying HNV grasslands with remotely sensed reflectance data [36], i.e., based on species communities rather than at the plant scale, significantly reduces the workload by excluding landscapes that lack HNV characteristics. Deep learning can then be applied to detect indicator plants on masked HNV grasslands. Linking different scales of landscape monitoring, e.g., identifying biodiversity hotspots using spectral information and examining identified hotspots in more depth using spatial information, is essential for successfully implementing biodiversity conservation actions. Additionally, this study holds the potential to be connected with results-based AES [48,49].

## CRediT authorship contribution statement

**Deepak H. Basavegowda:** Conceptualization, Data Curation, Formal Analysis, Investigation, Methodology, Project Administration, Resources, Software, Validation, Visualization, Writing - Original Draft. **Inga Schleip:** Conceptualization, Funding Acquisition, Methodology, Supervision, Writing - Review & Editing. **Paul Mosebach:** Conceptualization, Investigation, Writing - Review & editing. **Cornelia Weltzien:** Conceptualization, Funding Acquisition, Methodology, Project Administration, Supervision, Writing - Review & Editing.

## Declaration of competing interest

The authors declare that they have no known competing financial interests or personal relationships that could have appeared to influence the work reported in this paper.
